# Divergent disruptive effects of soluble recombinant tau assemblies on synaptic plasticity in vivo

**DOI:** 10.1186/s13041-025-01208-8

**Published:** 2025-04-18

**Authors:** Yin Yang, Tomas Ondrejcak, Neng-Wei Hu, Igor Klyubin, Michael J. Rowan

**Affiliations:** 1 Department of Physiology and Neurobiology, School of Basic Medical Sciences, Zhengzhou University, 100 Science Avenue, Zhengzhou, 450001 China; 2 Department of Pharmacology & Therapeutics, School of Medicine, Institute of Neuroscience, Watts Building, Trinity College, Dublin 2, Ireland

**Keywords:** Alzheimer’s disease, Recombinant tau, Tumor necrosis factor (alpha), Long-term potentiation, Long-term depression, Synaptic plasticity

## Abstract

Neurofibrillary tangles (NFTs), composed of tau protein fibrils, together with brain inflammation and synaptic loss, are neuropathological hallmarks of several neurodegenerative diseases, including Alzheimer’s disease. Compared with tau fibrils, more water-soluble assemblies of either recombinant or patient-derived tau have been reported to exert relatively potent rapid synaptotoxic effects, including inhibition of synaptic long-term potentiation (LTP) in the hippocampus. Less is known regarding the action of exogenous tau soluble assemblies on the opposite form of synaptic plasticity, long-term depression (LTD). We compared the synaptic plasticity actions of two relatively standard preparations of soluble recombinant tau assemblies, (i) fibril-derived soluble sonicated tau aggregates (SτAs) and (ii) oligomer-enriched tau (oTau) prepared from monomers. Consistent with previous findings, intracerebroventricular injections of either SτAs or oTau acutely inhibited high-frequency stimulation-induced LTP at CA3-to-CA1 synapses in the anaesthetized rat hippocampus. However, LTP inhibition by oTau, but not SτAs, was prevented by co-injection with the conformational anti-tau monoclonal antibody, TOMA1. Furthermore, in contrast to SτAs, which inhibited LTD, injection of oTau potently facilitated LTD, decreasing the threshold for LTD induction by low-frequency stimulation. To test the role of pro-inflammatory cytokines in mediating the disruptive effects of the two forms of soluble tau on synaptic plasticity we pre-injected etanercept, a decoy receptor for tumor necrosis factor alpha (TNFα). Etanercept reduced the disruption of synaptic plasticity by oTau but not by SτAs. Moreover, injection of exogenous TNFα mimicked the facilitation of LTD by oTau, consistent with a role of this pro-inflammatory cytokine in LTD facilitation.

These data provide evidence that preparations of soluble tau containing either monomer- or fibril-derived assemblies disrupt LTP and LTD via different mechanisms. Intriguingly, when oTau and SτAs were applied together, LTD block predominated. Thus, if similar synaptotoxic soluble tau assemblies are present together later during the disease process, as seems likely, inhibition of synaptic weakening processes is predicted to predominate. Equally, reducing TNFα would be expected to be more effective when the monomer-derived soluble tau assemblies are the dominant synaptotoxic species. We conclude that oTau and SτAs provide useful means to explore ways of targeting different synaptotoxic soluble tau species in tauopathies.

**Supplementary Information:**

The online version contains supplementary material available at 10.1186/s13041-025-01208-8.

## Introduction

Alzheimer’s disease (AD) is the most common cause of progressive dementia in the elderly. Currently more than 55 million individuals live with dementia worldwide, whereof AD is the most common form accounting for 60–70% of cases (https://www.alzint.org/about/dementia-facts-figures/dementia-statistics/). Neuropathological hallmarks of AD include extracellular deposits of amyloid beta (Aβ) peptides assembled in plaques, intraneuronal accumulation of misfolded tau protein forming neurofibrillary tangles (NFTs), and chronic brain inflammation. The severity of dementia in AD positively correlates with the number of NFTs, which are composed of insoluble tau fibrillar aggregates (paired helical filaments, PHFs) [1]. Interestingly, different tau assemblies can cause neurotoxicity, inflammation, and cognitive decline even without amyloid plaques, as seen in other tauopathies like Pick’s disease (PiD), progressive supranuclear palsy, and corticobasal degeneration [2]. Although NFTs are a hallmark of AD, neurons containing them are able to survive for years [3], suggesting that intracellular NFTs alone may not be the main tau species that cause neuronal damage. Indeed, abnormally aggregated tau fibrils modelling PHFs can sequester misfolded toxic tau in vitro [4], which might initially be neuroprotective [5, 6]. Conversely, NFTs may be a source of more soluble fibril-derived tau aggregates [7] that can act as seeds to spread tau pathology [7–9]. A growing body of research indicates that predominantly small, soluble forms of tau, known as tau oligomers, are more harmful than NFTs [8, 10–12]. These oligomeric forms can be released by neurons and may also act as seeds that spread tau pathology and impair cognition [13, 14].

Tau oligomers appear to play a major role in synaptic dysfunction, a key factor in AD-related cognitive decline [15]. Several studies have demonstrated that administration of different preparations of oligomeric tau (synthetic, from transgenic mice or from AD brains) impaired synaptic plasticity and memory [8, 10, 13, 16–18]. The role of soluble oligomers also emerged in studies performed in transgenic AD rodent models, since synaptic and memory dysfunction was present before the appearance of tangles [19].

Elevated levels of several different tau species in samples from AD patients have been associated with disease severity, highlighting their potential significance in the development of AD and related disorders [20–22]. Recent data indicate that multiple distinct bioactive tau species co-occur within the same AD brain, supporting the idea that multiple tau conformers, both fibrillar and nonfibrillar, can differently impact phenotype in AD [23]. A striking heterogeneity of soluble oligomeric tau species have been isolated from human tauopathy brains that were associated with diverse tau neuropathology and symptom severity [24–26]. These findings highlight the possibility that AD involves multiple biochemically distinct tau assemblies that underpin disease progression with various symptoms/manifestations and subtypes. In this context, significant advances have been made in the development of novel anti-tau antibodies that target unique conformational epitopes, independent of specific linear amino acid sequences, and exhibit preferences for different subsets of tau assemblies [9, 27, 28].

Pro-inflammatory processes have recently emerged as an important component of early AD pathogenesis [29–32]. A particularly attractive therapeutic strategy is to selectively prevent the disruptive effects of activation of the innate immune system in the brain at an early stage by reducing the production or directly neutralizing pro-inflammatory cytokines, particularly interleukin-1 beta and tumor necrosis factor alpha (TNFα). Several lines of evidence using genetic and pharmacological manipulations indicate that TNFα signaling exacerbates both Aβ and tau pathologies in vivo [33]. Interestingly, anti-inflammatory strategies can reduce brain pathology and improve cognitive function in rodent models of AD [34]. Clinical trials exploring the efficacy of anti-TNFα drugs on the progression of AD [35, 36] are ongoing.

Here, we examined the ability of two different soluble preparations of full-length 2N4R recombinant tau, fibril-derived soluble sonicated tau aggregates (SτAs) and monomer-derived oligomer-enriched tau (oTau), on synaptic plasticity in the anaesthetised rat hippocampus. Whereas both preparations inhibited LTP, they had opposite effects on LTD. Moreover, the conformational anti-tau monoclonal antibody (mAb), TOMA1 and the TNFα decoy receptor etanercept only attenuated the disruptive actions of oTau.

## Methods

### Animals, cannula and electrode implantation, and sample injection procedure

All experiments were conducted in accordance with ARRIVE (Animal Research: Reporting of In Vivo Experiments) guidelines under the approval of Trinity College Dublin local animal research ethics committee and the Health Products Regulatory Authority in Ireland, using methods similar to those described previously [37]. Food and water were available *ad libitum* with a 12 h light/dark cycle. In the study, we used 1.5-4-month-old male Lister Hooded (LH) rats. We previously reported that synaptic plasticity in LH rats is very sensitive to the disruptive action of disease relevant tau species [38, 39].

**Fig. 1:**
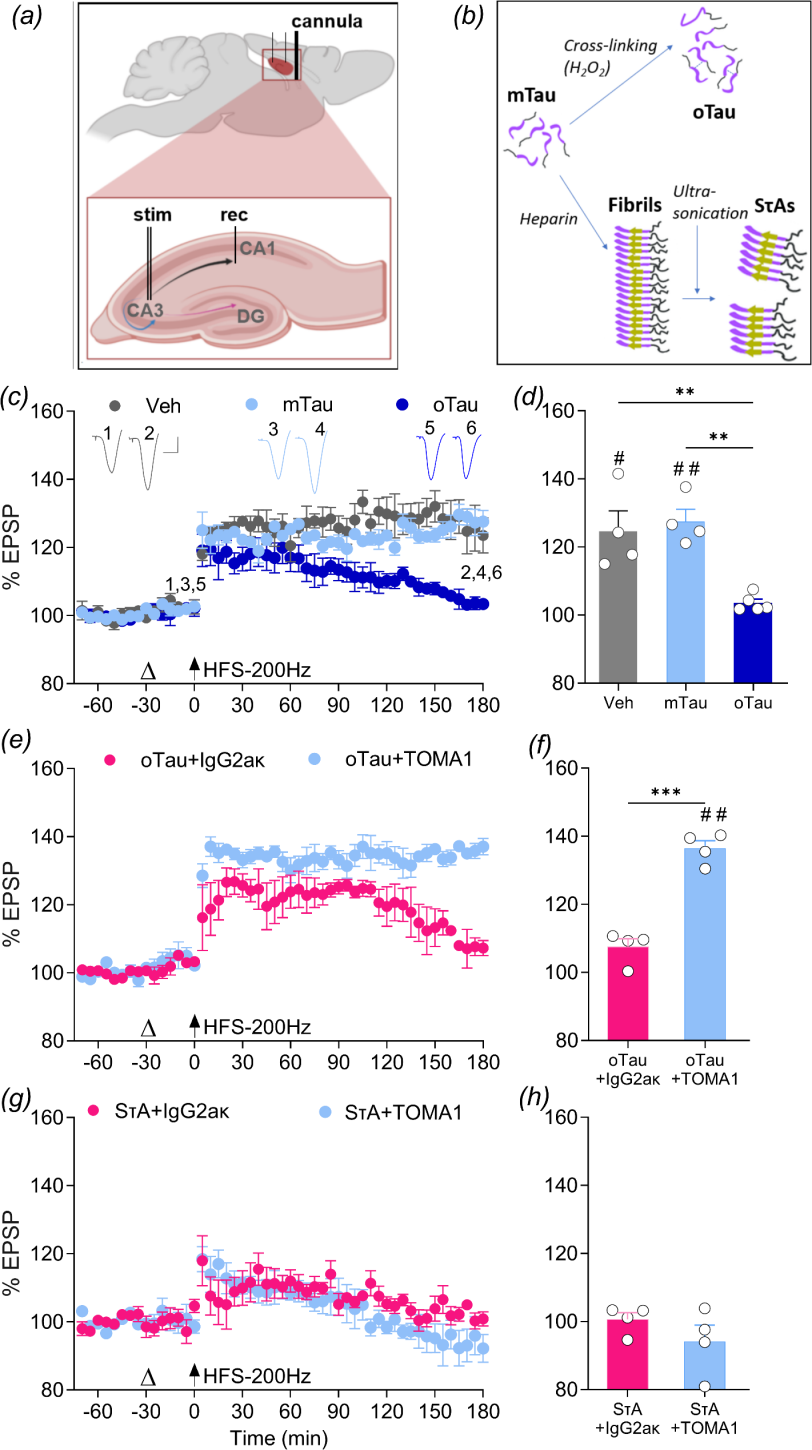
The conformational tau antibody TOMA1 prevents LTP inhibition by oligomer-enriched tau preparation (oTau) but not soluble sonicated tau aggregates (SτAs) in vivo. (**a**) Schematic diagram of in vivo electrophysiological methods used to record synaptic plasticity in the hippocampal network of urethane-anesthetized rats. Field evoked postsynaptic potentials (EPSPs) were electrically evoked in the Schaffer collateral/commissural pathway by electrically stimulating (“stim” electrode) apical dendrites in the CA3 area and recording EPSPs (using “rec” electrode) in the CA1 area of the hippocampus. DG– dentate gyrus. (**b**) Schematic illustration of two different recombinant tau aggregation protocols where the same starting material, monomeric human WT hTau40 (mTau), was used to produce either cross-linked tau oligomers (oTau), or soluble sonicated tau fibril-derived aggregates (SτAs), see methods for more details. (**c**,** d**) Acute i.c.v. injection (open triangle) in urethane anaesthetised rats of 4.9 pmol oTau inhibited standard conditioning stimulation (200 Hz train, HFS; arrow)-induced LTP. In contrast, HFS triggered robust LTP in rats injected with this dose of tau monomers (mTau). Vehicle-injected animals (Veh) served as interleaved controls. (**e**,** f**) Whereas oTau (4.9pmol) also inhibited HFS-induced LTP when co-injected with an isotype control antibody (oTau + IgG2ak) it failed to do so in anti-tau antibody (oTau + TOMA1) co-treated rats. **(g**,** h)** I.c.v. injection of 1.2 pmol SτAs inhibited LTP when co-injected with either the conformational anti-tau antibody (SτAs + TOMA1, 2.5 µg) or the isotype control antibody (SτAs + IgG2ak, 2.5 µg). Summary bar charts in **d**,** f** and **h** show the magnitude of LTP during the last 10 min for the time-course data in **c**,** e** and **g**, respectively. Representative EPSP traces at 10 min prior to HFS and 180 min post-HFS. Scale bars: vertical, 1 mV; horizontal, 10 ms. Values are mean ± SEM. ^#^*p* < 0.05, ^##^*p* < 0.01 compared with pre-HFS baseline, paired *t*-test; **p < 0.01 one-way ANOVA followed by Bonferroni’s multiple-comparison tests (for comparison between 3 groups), or ***p < 0.001, unpaired t-test (to compare 2 groups)

To inject samples and test synaptic plasticity, the animals were anaesthetized with urethane (1.6 g/kg, i.p.) and core body temperature was maintained at 37.5 ± 0.5 °C. An intracerebroventricular (i.c.v.) stainless steel guide cannula (22 gauge, 0.7 mm outer diameter, length 13 mm) was implanted above the right lateral ventricle (coordinates, 0.5 mm posterior to bregma and 1.2 mm right of midline, depth 4 mm) before the electrodes were implanted ipsilaterally (a schematic diagram of cannula and electrode configuration is shown in Fig. 1a). Teflon-coated tungsten wire (external diameter 75 μm bipolar or 112 μm monopolar) electrodes were positioned in the stratum radiatum of area CA1. The electrodes were optimally located using a combination of physiological and stereotactic indicators (3.8 mm posterior to bregma and 2.5 mm lateral to midline, and 4.6 mm posterior to bregma and 3.8 mm lateral to midline for recording and stimulating electrodes, respectively). Screw electrodes located over the contralateral cortex were used as reference and earth. To inject samples acutely, a Hamilton syringe was connected to the internal cannula (28 gauge, 0.36 mm outer diameter). The injector was removed 1 min post-injection and a stainless-steel plug was inserted. All experiments were conducted under non-recovery anaesthesia using urethane, and animals were humanely euthanized at the end of the experiment. Verification of the placement of cannula was performed post-mortem by checking the spread of ink dye.

### Preparation of recombinant tau samples

#### Oligomer-enriched tau (oTau)

Oligomers of recombinant wild-type full-length human tau-441 (2N4R, MW = 45.9 kDa) used in these studies were previously prepared and described in [18, 40]. Briefly, human recombinant tau (R&D, #SP-495) solution with a starting concentration of 2 mg/ml was initially treated with the reducing agent TCEP (final concentration 1 mM, Thermo Scientific, #20490) and 5 mM EDTA (Millipore, #324506) at 37 °C for 1 h. Following the reduction step, TCEP and EDTA were eliminated using a 10K MWCO protein concentrator (Thermo Scientific, #88513) to prepare sample of tau monomers (mTau) The tau protein was then oligomerized by oxidative cross-linking with 1 mM H_2_O_2_ at room temperature for 20 h. Next, we performed buffer exchange to remove excess chemicals in a refrigerated centrifuge (12,000 g, 4 °C). Aliquots of tau solution were immediately frozen on dry ice and stored at -80 °C.

#### Soluble sonicated tau aggregates (SτAs)

We investigated the effects of recombinant, full-length human wild-type sequence tau expressed, purified and monomer concentrated in Dr Dominic Walsh’s lab as described and used previously [38–40]. Heparin was then added to promote aggregation and fibrils harvested by ultracentrifugation. Tau fibrils were ultrasonicated to prepare SτAs.

The preparations of tau monomers, tau oligomers and SτAs (schematic diagram of preparation methods shown in Fig. 1b) used in this study were derived from batches prepared and previously described in [18]. These batches were previously imaged by atomic force microscopy (AFM) [18]. The AFM average maximum heights of particles were 0.9 ± 0.7, 1.7 ± 2.6, and 2.5 ± 4.8 nm for mTau, oTau and SτAs, respectively.

### Compounds and antibodies

Etanercept (commercially sold as Enbrel^©^), a soluble dimeric form of the TNF receptor that very selectively binds and neutralizes TNF [41], was purchased from the Pharmacy Department, St. James’s Hospital, Dublin, Ireland. TOMA1 (Millipore; #MABN819), a mouse monoclonal antibody targeting tau oligomers [8] and isotype control monoclonal antibody (mouse IgG2aκ clone eBM2a, Invitrogen; #14-4724-85) were used. TNFα (Sigma; #GF442-M) was prepared in distilled water.

The route, dose and timing choices for injection of the different agents were based on pilot experiments and previous reports. Doses of SτAs (up to 2.8 pmol, i.c.v) that inhibited LTP previously did not affect baseline synaptic transmission [39]. In pilot studies the injection of the higher dose of oTau (12.3 pmol, i.c.v.), without the application of LFS protocol, had no observable effect on baseline synaptic transmission (oTau-baseline: 104.5 ± 7.7% at 3.5 h post-injection, *n* = 4; *p* = 0.5327 vs. pre-injection, paired t test). Similar to SτAs [39], this dose of oTau did not affect paired-pulse facilitation at a 40 ms inter-pulse interval (96.9 ± 4.5% baseline facilitation at 3.5 h post-injection, *n* = 4; *p* = 0.6289 vs. pre-injection baseline, paired t test). For co-injection of antibodies, the recombinant tau preparations were co-incubated for 5 min prior to i.c.v. injection using doses based on prior findings [18, 38, 39]. Etanercept was pre-injected (i.c.v.) 15 min prior to or simultaneously with the injection of recombinant tau. We found no apparent difference between the two methods of administration. The dose of etanercept (50 µg in 5 µL, i.c.v.) used here was previously reported to restore disrupted LTP in freely-moving transgenic rats overexpressing AD-associated β-amyloid precursor protein at a pre-plaque stage of amyloidosis [42]. The dose of TNFα (1.5 pmol) is based on our previous report [43] showing acute inhibition of LTP when pre-injected (i.c.v.) 30 min before conditioning stimulation.

### In vivo electrophysiology

CA3-to-CA1 field excitatory postsynaptic potentials (EPSPs) were evoked and recorded in the stratum radiatum under urethane anaesthesia. LabChart 7 software controlled an analogue-to-digital system consisting of an in-house pre-amplifier (broadband range up to 4 kHz) connected to a PowerLab 2/26 (ADInstruments, Australia) for data acquisition and analysis. Single square-wave pulses (0.2 ms duration) generated by a constant current isolation unit PSIU6 (Grass Instruments Co., USA) were applied every 30 s at intensity that triggered a 50% maximum EPSP response. Paired-pulse facilitation (PPF) was tested using two stimuli using the test pulse intensity at a 40 ms interval. The magnitude of PPF was calculated as the ratio of amplitude of the second EPSP to the first, and then expressed as a % of the pre-injection baseline. To induce NMDA receptor-dependent LTP standard 200 Hz conditioning stimulation protocol (HFS-200 Hz), consisting of one set of 10 trains of 20 pulses (inter-train interval of 2 s) at test intensity, was applied. A peri- and supra-threshold low-frequency electrical stimulation (LFS) protocols, used to study the tau-mediated disruption of LTD [44], consisted of either 300 or 900 pulses (0.2 ms duration) at 1 Hz, with an intensity that evoked 95% maximum amplitude. To minimize the possible confounding effect of variation in control LTP and LTD, the experiments in each study were interleaved. There were no detectible abnormal changes in background hippocampal EEG which was monitored throughout the experiments.

### Data analysis

Values are presented as the mean ± SEM percentage pre-injection baseline EPSP amplitude over a 45 min period. The magnitude of LTP/LTD was measured at 3 h post-conditioning stimulation and expressed as the mean ± SEM % baseline. For graphing purposes, EPSP amplitude measurements were grouped into 5 min (average of 10 sweeps). For statistical analysis, data are expressed as the average EPSP amplitude during the last 10 min epoch before and 170–180 min (3 h) after HFS/LFS. Sample sizes were chosen based on our previous publications with similar experimental designs [45].

The ability to induce LTP and LTD within each experimental group was assessed a priori using paired two-tailed *t* tests. Differences in the magnitude of potentiation/depression between experimental groups were analysed using one-way ANOVA with Bonferonni’s *post hoc* tests or by unpaired two-tailed *t* tests, as appropriate. A *p* value of < 0.05 was considered statistically significant. Statistical analyses were performed in GraphPad Prism software (10.3.1).

## Results

### Conformation-selective antibody prevents LTP inhibition by oTau but not SτAs

In the first sets of experiments, we tested the LTP disruptive actions of two different preparations of synaptotoxic recombinant full-length tau aggregates: (i) oTau prepared directly from tau monomers (mTau) [16, 18] and (ii) soluble sonicated tau aggregates (SτAs) prepared from pre-formed tau fibrils [39, 40] (Fig. 1b). Consistent with previous reports of the acute LTP disruptive action of oTau in vitro [17] and in vivo [18], the application of HFS-200 Hz 30 min after i.c.v. injection of oTau (4.9 pmol, i.c.v.) only induced a decremental LTP (oTau: 103.6 ± 1.1% at 3 h post-HFS; *p* = 0.6779 compared with pre-HFS baseline, paired t test; *n* = 5; F_(2,10)_ = 13.10, *p* = 0.0016, 1-W-ANOVA; *p* = 0.0069 vs. Vehicle, Bonferroni post-hoc test) whereas it induced robust LTP when applied after injecting the same dose of tau monomers (mTau: 127.5 ± 3.6% at 3 h post-HFS; *p* = 0.0036 compared with pre-HFS baseline, paired t test; *n* = 4) or vehicle (Veh: 124.7 ± 6%, *n* = 4) (Fig. 1c, d). The lack of effect of mTau on LTP is consistent with our previous report [39].

Because oTau mimics the LTP inhibitory effect of SτAs [37–39] we wondered if the same conformation of synaptotoxic tau was responsible. To test this idea, we co-incubated either oTau or SτAs with TOMA1, a conformation-selective antibody generated against tau oligomers [8] or an isotype control antibody. In the case of co-injection of oTau (4.9pmol, i.c.v.) and TOMA1 (2.5 µg), the application of HFS-200 Hz induced robust LTP (oTau + TOMA1: 136.5 ± 2.3% at 3 h post-HFS; *p* = 0.0029 compared with pre-HFS baseline, paired t test; *n* = 4; *p* = 0.0001 unpaired t test, compared to IgG2ak + oTau: 107.5 ± 2.4% elicited in isotype control-injected rats, *n* = 4, *p* = 0.0973 paired t test vs. pre-HFS baseline) (Fig. 1e, f).

In the case of SτAs we used a dose of 1.2 pmol i.c.v. that strongly inhibits LTP [37–39]. In contrast to oTau, after co-injection of SτAs with TOMA1 (2.5 µg, i.c.v.) HFS failed to induce persistent LTP (TOMA1 + SτAs: 94.10 ± 4.8% at 3 h post-HFS; *p* = 0.3222 compared with pre-HFS baseline, paired t test; *n* = 4; *p* = 0.2622, unpaired t test), similar to SτAs co-injected with the isotype control antibody (IgG2ak + SτAs: 100.6 ± 2.1% at 3 h post-HFS; *p* = 0.7558 compared with pre-HFS baseline, paired t test; *n* = 4)(Fig. 1g, h).

These findings indicate that the synaptotoxic tau species in oTau and SτA preparations are different.

### An anti-TNFα agent prevents LTP inhibition by oTau but not SτAs

**Fig. 2:**
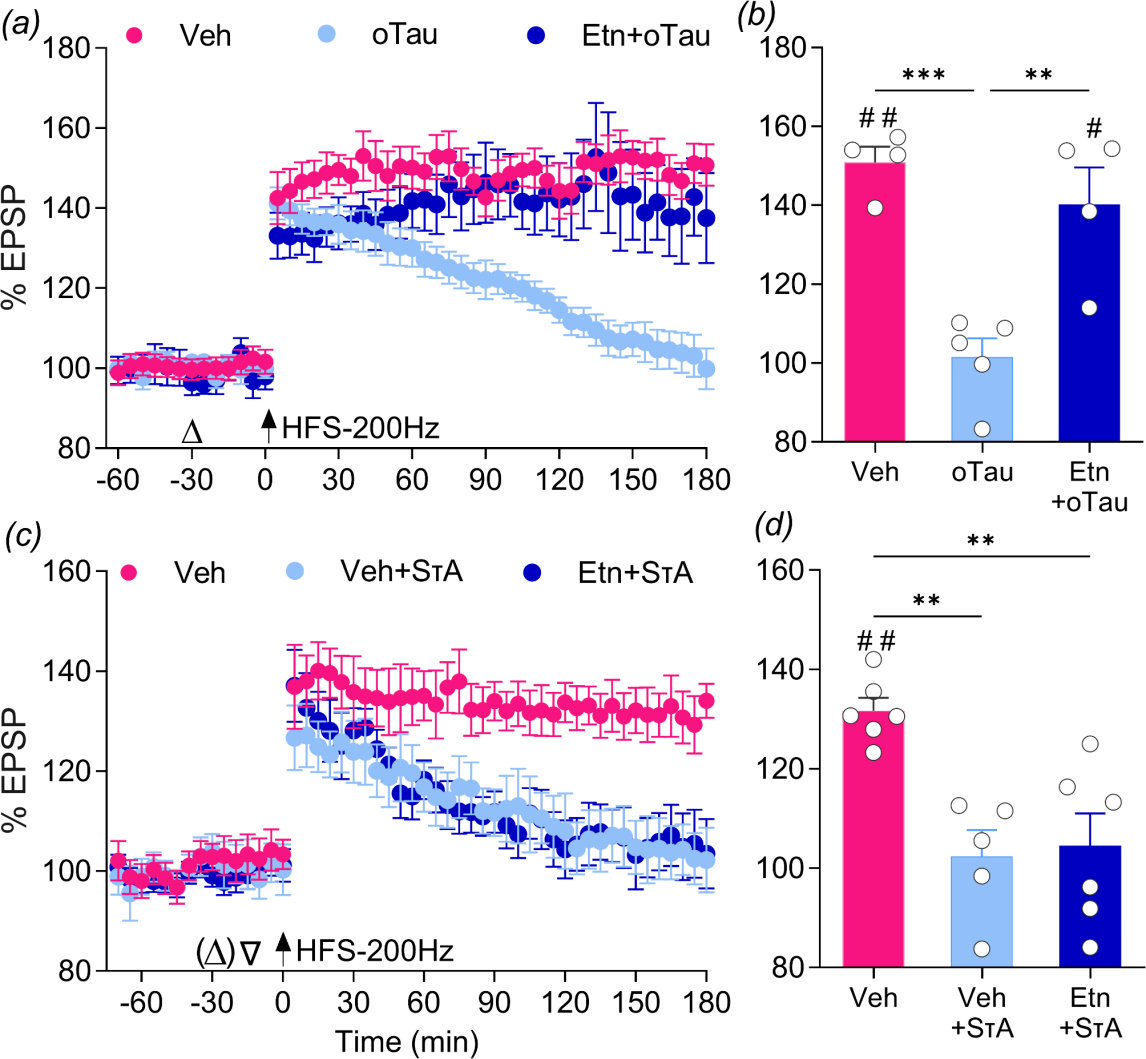
The TNFα inhibitor etanercept abrogates disruption of hippocampal LTP caused by oTau but not SτAs. (**a**,** b**) Co-injection of etanercept (Etn, 50 µg, i.c.v.) prevented the inhibition of LTP by i.c.v. injection of synaptotoxic oTau (12.3 pmol). Open triangle: injection of Vehicle, oTau or Etanercept + oTau. (**c**,** d**) Injection of etanercept (Etn, 50 µg, i.c.v.) did not prevent the inhibition of LTP caused by i.c.v. injection of SτAs (1.2 pmol, *n* = 4 pre-injected and *n* = 2 co-injected). Open triangle: injection of either Vehicle, SτAs or Etn co-injected with SτAs. Open triangle in parentheses: in a subset of experiments Etn was pre-injected 15 min before SτAs. Values are mean ± SEM. ^#^*p* < 0.05, ^##^*p* < 0.01 compared with pre-HFS baseline, paired *t*-test; **p < 0.01, ***p < 0.001 one-way ANOVA followed by Bonferroni’s multiple-comparison tests

The neuroinflammatory processes in AD are mainly driven by the innate immune cells in the brain, including microglia and astrocytes [46]. A diverse range of tau assemblies can trigger an increase in pro-inflammatory cytokines, including the master regulator of inflammation, TNFα [30, 47]. Previously, we reported that etanercept, a decoy receptor for TNFα, prevented LTD facilitation by AD brain soluble synaptotoxic tau [48]. To determine if the inhibition of LTP by synaptotoxic oTau was mediated by this cytokine, we used a similar strategy. Whereas the application of 200 Hz HFS in rats injected 30 min previously with oTau (12.3 pmol, i.c.v.) induced a decremental LTP (101.4 ± 4.9% at 3 h post-HFS; *p* = 0.8246 compared with pre-HFS baseline, paired t-test; *n* = 5; vs. Vehicle: 150.9 ± 4%, *n* = 4, *p* = 0.0014, paired t-test; F_(2,10)_ = 17.85, *p* = 0.0005, 1-W-ANOVA; *p* = 0.0007 for Veh vs. oTau, Bonferroni post-hoc test), in rats co-injected with etanercept (50 µg, i.c.v.) robust LTP was induced (Etn + oTau: 140.2 ± 9.5% at 3 h, *n* = 4, *p* = 0.0284, paired t-test; *p* = 0.0041 and 0.8338 when compared vs. oTau or Veh, respectively, Bonferroni post-hoc tests) (Fig. 2a, b).

Consistent with our previous report [39], LTP was strongly inhibited after acute i.c.v. injection of 1.2 pmol dose of SτAs (Veh + SτAs: 102.4 ± 5.3% at 3 h, *n* = 5, *p* = 0.8521, paired t-test). Notably, injection of etanercept (50 µg, i.c.v.) with SτAs failed to abrogate the LTP inhibition (Etn + SτAs: 104.5 ± 6.45% at 3 h, *n* = 6, *p* = 0.5625 compared with pre-HFS baseline, paired t-test; vs. Veh: 131.7 ± 2.6% at 3 h, *n* = 6, *p* = 0.0013, paired t-test; F_(2,14)_ = 10.54, *p* = 0.0016, 1-W-ANOVA; *p* = 0.0040 and 0.0049 for Veh vs. SτAs or Veh vs. Etn + SτAs, respectively, Bonferroni post-hoc test)(Fig. 2c, d**).**

### An anti-TNFα agent prevents oTau-mediated LTD facilitation but not SτA-mediated LTD inhibition

**Fig. 3:**
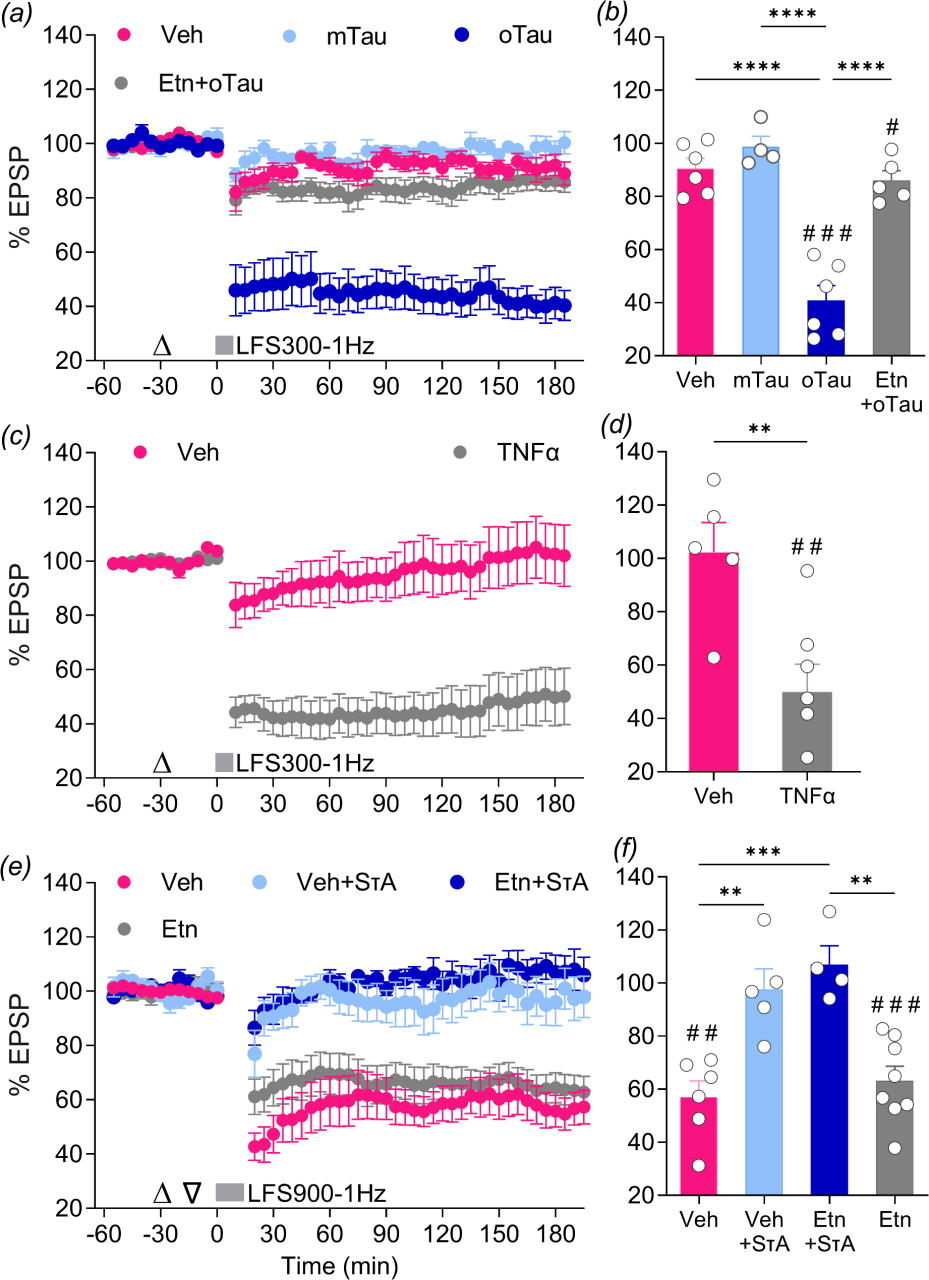
The TNFα inhibitor etanercept abrogates disruption of hippocampal LTD caused by oTau but not SτAs. (**a**,** b**) The application of weak LFS (bar, LFS300-1 Hz, 300 pulses at 1 Hz) did not induce stable LTD in rats treated with either vehicle (Veh) or tau monomers (mTau, 12.3 pmol). In contrast, acute i.c.v. injection of oTau (12.3 pmol) enabled the induction of a robust and stable LTD by LFS300-1 Hz. Etanercept (Etn, 50 µg/5 µL, i.c.v.) prevented the facilitation of LTD by i.c.v. administration of oTau, 12.3 pmol (Etn + oTau). (**c**,** d**) Injection of the pro-inflammatory cytokine TNFα (1.5 pmol, i.c.v.) also facilitated the induction of stable LTD by LFS300-1 Hz compared with vehicle-treated rats (Veh). (**e**,** f**) The application of strong LFS (bar, LFS900-1 Hz, 900 pulses at 1 Hz) induced stable LTD in anaesthetized rats treated with vehicle (Veh, i.c.v.) or etanercept only (Etn, 50 µg). Acute i.c.v. injection of SτAs (1.2 pmol) inhibited the induction of LTD by LFS900-1 Hz (Veh + SτAs), an effect that was not prevented by pre-treatment with etanercept (Etn + SτAs). Values are mean ± SEM. ^#^*p* < 0.05, ^##^*p* < 0.01, ^###^*p* < 0.001 compared with pre-LFS baseline, paired *t*-test; **p < 0.01, ***p < 0.001 or ****p < 0.0001 one-way ANOVA followed by Bonferroni’s multiple-comparison tests, or **p < 0.01 unpaired t test

Our recent report [48] suggests that soluble synaptotoxic tau in AD brain extracts facilitates LTD whereas SτAs potently inhibit LTD [38]. We therefore decided to determine if oTau facilitated LTD. We used a relatively weak LFS conditioning protocol, 300 high-intensity pulses at 1 Hz (LFS300-1 Hz), that was just below the threshold for inducing stable hippocampal LTD in vivo. Thus, application LFS300-1 Hz failed to induce stable LTD at CA3-to-CA1 synapses in adult anaesthetized rats injected with Vehicle (Veh: 90.5 ± 3.9%, *n* = 6, *p* = 0.11, paired t-test compared to pre-LFS values) (Fig. 3a, b). In contrast, this weak LFS300-1 Hz protocol induced stable and robust LTD after i.c.v. injection (12.3 pmol) of oligomer-enriched preparation of recombinant tau (oTau: 40.8 ± 5.6%, *n* = 6, *p* = 0.0001, paired t-test compared to pre-LFS baseline; F_(3,17)_ = 34.73, *p* < 0.0001, 1-W-ANOVA; *p* < 0.0001 oTau vs. Veh, Bonferroni post-hoc test), but not using the same dose of tau monomers (mTau: 98.8 ± 3.8%, *n* = 4, *p* = 0.44, paired t-test) (Fig. 3a, b).

Because TNFα appears to mediate LTD facilitated by soluble synaptotoxic tau in AD brain extracts [48], we hypothesized that TNFα would be necessary for oTau-facilitated LTD. Indeed, pre-injecting etanercept (50 µg, i.c.v.) before oTau (12.3 pmol) prevented oTau-facilitated LTD (Etn + oTau: 86.1 ± 3.7%, *n* = 5, *p* = 0.0202, paired t-test; *p* > 0.9999 and *p* < 0.0001 for Etn + oTau vs. Veh or vs. oTau, respectively, Bonferroni post-hoc tests)(Fig. 3a, b**)**. To determine if TNFα alone was sufficient to facilitate LTD we injected a dose of TNFα (1.5 pmol, i.c.v.) that inhibits LTP in vivo [43]. TNFα mimicked the effect of oTau on LTD induced by LFS300-1 Hz (TNFα: 50 ± 10.4% at 3 h post LFS300-1 Hz, *n* = 7, *p* = 0.0028, paired t-test compared to pre-LFS baseline; *p* = 0.0071 vs. Veh: 102 ± 11.4%, *n* = 5, unpaired t test) (Fig. 3c, d**).** These data suggest that TNFα could serve as a critical mediator of LTD-facilitating effects of oTau.

In order to determine if TNFα mediates the inhibition of LTD by SτAs we used the standard strong LFS protocol, LFS900-1 Hz. As previously reported by our group [38], injection of SτAs (1.4pmol, i.c.v., 15 min before LFS900-1 Hz) strongly inhibited LTD when compared with vehicle-injected animals (Veh + SτAs:97.6 ± 7.8% measured at 3 h post LFS, *n* = 5, *p* = 0.5133, paired t-test compared to pre-LFS baseline; vs. Veh: 57 ± 6.1%, *n* = 6, *p* = 0.0024, paired t-test; F_(3,19)_ = 13.13, *p* < 0.0001, 1-W-ANOVA; *p* = 0.0024 for Veh + SτAs vs. Veh, Bonferroni post-hoc test) (Fig. 3e, f). Interestingly, and similar to LTP inhibition by SτAs, LTD inhibition mediated by SτAs was not prevented by i.c.v. pre-injection of etanercept, using the same dose (50 µg) that attenuated oTau-facilitated LTD (Etn + SτAs:107 ± 7.1%, *n* = 4, *p* = 0.3191, paired t-test compared to pre-LFS baseline; *p* = 0.0005 and > 0.9999 vs. Veh or Veh + SτA, respectively, Bonferroni post-hoc) (Fig. 3e, f). Etanercept on its own had no effect on the induction of LTD by LFS900-1 Hz (Etn: 63.1 ± 5.5%, *n* = 8, *p* = 0.0003, paired t-test; *p* > 0.9999 vs. Veh, Bonferroni post-hoc test). In conclusion, neither LTP nor LTD inhibition by SτAs was alleviated by etanercept (Figs. 2c and d and 3e and f**)**.

### SτAs prevent oTau-mediated LTD facilitation

**Fig. 4:**
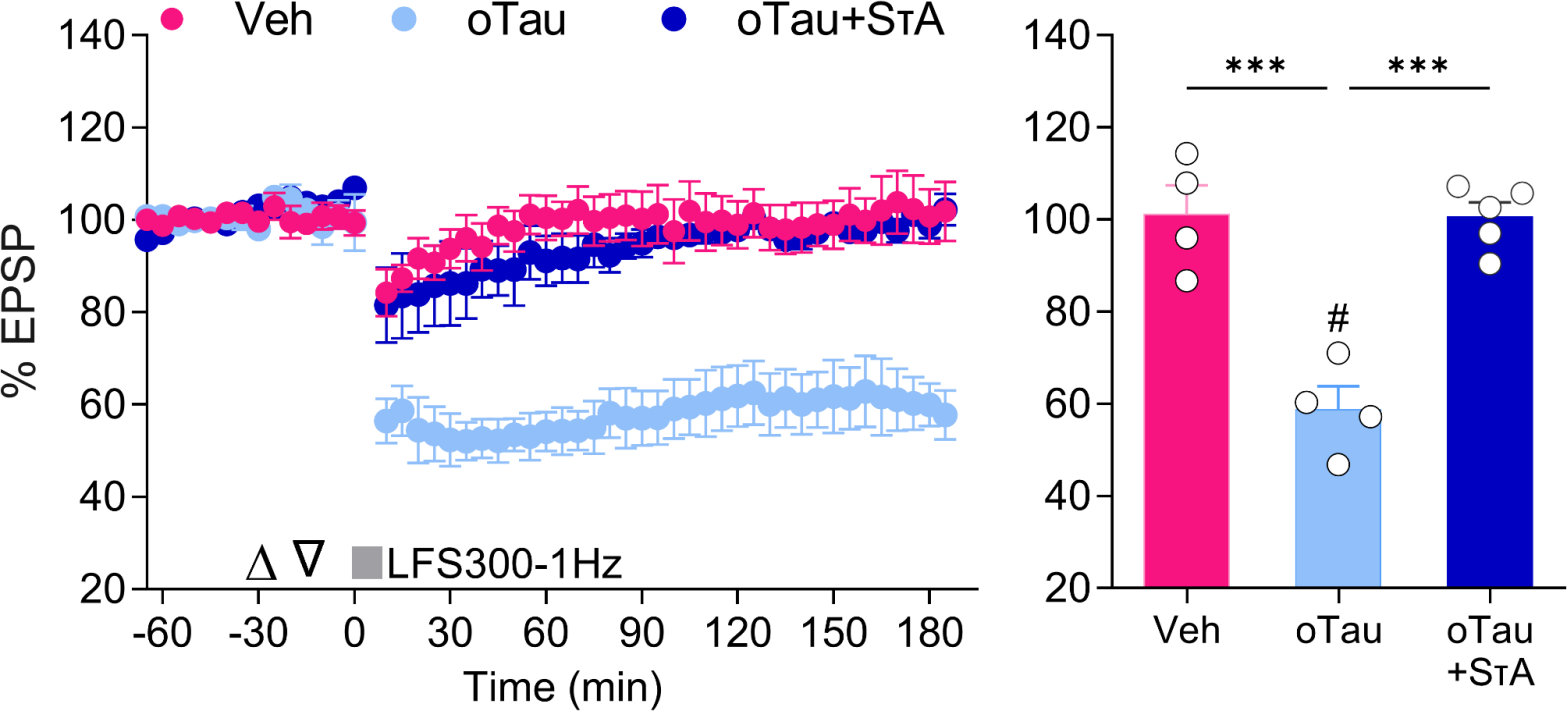
SτAs prevent oTau-facilitated LTD. Animals received two separate i.c.v. injections (open triangles) either vehicle (Veh), oTau (12.3 pmol, a dose that facilitates LFS300-1 Hz-induced LTD) or oTau and SτAs (0.6 pmol, a dose that blocks LFS300-1 Hz-induced LTD) with the injection sequence roughly counterbalanced (oTau + SτAs, three animals receiving SτAs first and the other two getting oTau first) before applying the weak induction protocol, LFS300-1 Hz. Values in right hand panel at 3 h post-LFS from left hand panel. Values are mean ± SEM. ^#^*p* < 0.05 compared with pre-LFS baseline, paired *t*-test; ***p < 0.001 one-way ANOVA followed by Bonferroni’s multiple-comparison tests

Given the opposite effects of two studied forms of recombinant tau on LTD, i.e. oTau causing facilitation and SτAs having an inhibitory effect, we asked which action predominates when both synaptotoxic species are present together. Using the relatively weak conditioning protocol (LFS300-1 Hz) we confirmed our finding that oTau (12.3 pmol, i.c.v.) facilitated LTD (Veh + oTau: 58.9 ± 5%, *n* = 4; *p* = 0.0138 compared with pre, paired t test; F_(2,10)_ = 25.99, *p* = 0.0001, 1-W-ANOVA; *p* = 0.003 Bonferroni post-hoc test vs. Veh: 101.3 ± 6.1%, *n* = 4)(Fig. 4). In contrast, LFS300-1 Hz failed to induce LTD in animals receiving a separate injection of SτAs (0.6 pmol, a dose that strongly blocks Aß oligomer-facilitated LFS300-induced LTD, see [38]) in addition to oTau, regardless of injection sequence (oTau + SτAs: 100.6 ± 3.1%, *n* = 5, three animals receiving SτAs first and the other two getting oTau first; *p* = 0.2491 compared with pre, paired t test; *p* > 0.9999 compared with Veh group and *p* < 0.0002 vs. Veh + oTau, Bonferroni post-hoc tests) **(**Fig. 4**)**. These findings indicate that the ability of oTau to lower the LTD induction threshold is overridden by SτA-mediated LTD inhibition. In order to establish the generality of the antagonism of oTau’s effect by SτAs, we decided to test the effect of SτAs on TNFα-facilitated LTD. Co-injection of the same dose of SτAs that attenuated oTau-facilitated LTD (0.6 pmol, see Fig. 4) and the dose of TNFα that facilitated LTD (1.5 pmol, see Fig. 3c, d) failed to induce LTD (SτAs + TNFα: 97.8 ± 6.2% at 3 h post LFS300-1 Hz, *n* = 3, *p* = 0.8775, paired t-test compared to pre-LFS baseline) (Supplemental Fig. 1, Additional file 1).

## Discussion

In this study, we compared the synaptic plasticity-disrupting effects of two preparations of recombinant tau (SτAs and oTau) that are used to model soluble tau assemblies in the brains of people with tauopathies, including AD. SτAs, derived from pre-formed fibrils and oTau prepared from monomers by inducing disulfide bonds exerted different actions on synaptic plasticity in the rodent hippocampus. Although both SτAs and oTau acutely inhibited LTP at CA3-to-CA1 synapses, only the disruptive effect of the latter tau preparation was attenuated by the conformational anti-tau antibody TOMA1 and the TNFα inhibitor etanercept. LTD was also inhibited by SτAs, but again, in an apparently TNFα-independent manner. Conversely, oTau lowered the threshold for the induction of LTD at hippocampal synapses, an effect that was reduced by etanercept and mimicked by TNFα, consistent with a role for TNFα in LTD facilitation. Thus, the dominant synaptotoxic assemblies in the SτA and oTau preparations are very different, as witnessed by their opposite effects on LTD and differential sensitivity to a conformational anti-tau antibody and the different reliance on the pro-inflammatory cytokine TNFα.

Over the last decade, numerous studies including ours demonstrated that exogeneous application of soluble tau assemblies can cause rapid and lasting impairment of synaptic function, suggesting a significant role in mediating synaptotoxicity in several neurological illnesses, including AD and other tauopathies. Indeed, both oTau [16, 18] and SτAs [39, 40] are known to mimic the ability of synaptotoxic tau in aqueous extracts of AD and PiD brain [16, 37, 40] to inhibit LTP. How well the responsible tau species in recombinant tau preparations model those in patient-derived samples is a matter of ongoing research. It is also unclear whether or not the same tau species that inhibit LTP also disrupt LTD. Synaptotoxic tau in AD and PiD aqueous brain extracts, which is mainly intracellular in origin, facilitates LTD [48]. We also reported [48] that similar lowering of LTD threshold is caused by extracellular synaptotoxic tau in secretomes of induced pluripotent stem cell-derived neurons from individuals with trisomy of chromosome 21 (Ts21) a common cause of early onset AD [49]. Here, we report that oTau mimics the LTD facilitating action of soluble synaptotoxic tau present in patient-derived aqueous brain extracts. In contrast, the present studies confirmed our previous report that SτAs potently inhibit LTD [38]. The opposite effects of SτAs and oTau on LTD strongly indicate that different synaptotoxic species are present in these two recombinant tau preparations.

In the present research full-length, unphosphorylated recombinant tau was polymerized using two different relatively standard methods. On the one hand, heparin was used to promote self-assembly of tau into fibrils that were subsequently broken down into smaller particles (SτAs) by ultrasonication [38, 40]. On the other hand, H_2_O_2_-mediated oxidative cross-linking of disulfide bonds between monomers was the source of the oTau preparation [16]. We and others previously reported that many distinct tau assemblies are present in the oTau and SτA preparations used in the present experiments [16, 18, 40, 50, 51]. Using AFM, we [18] and others [16] found found that oTau appears as punctate structures, slightly larger than monomers. This is consistent with previous AFM observations of disease-relevant brain-derived tau oligomers [16, 50, 51] indicating the presence of classical homogeneous spherical morphology [51]. Using western blotting Fa *et* al. [16] determined that the tau assemblies seen with AFM in oTau preparations are predominantly low-n oligomers. Although AFM of the SτAs used here include morphologically distinct non-filamentous aggregates, transmission electron microscopy analysis of the same SτA preparation [38] reveals a mixture of species, including imperfect spheres and abundant short filaments. Based on these considerations, it seems likely that the synaptic plasticity-disrupting tau species in the oTau preparation is a low-n oligomeric assembly whereas it is unclear which of the many other, presumably larger tau assemblies is responsible for the synaptotoxicity of SτAs.

Because of their ability to distinguish between different aggregation states relatively independent of primary amino acid sequence, conformational antibodies provide a complementary means of characterising tau assemblies mediating synaptic plasticity disruption in the present in vivo studies. The anti-tau antibody TOMA1 was generated using recombinant oligomeric tau (prepared by cross-seeding with synthetic Aß oligomers) as an immunogen [11] and was reported not to recognize monomeric tau or mature meta-stable NFTs [8]. Single or long-term administration of this mAb had beneficial effects in several murine models of tau deposition ( [8], but see [52]), inhibiting oligomeric tau accumulation and preserving memory function. Our finding that TOMA1 prevented the LTP inhibition by oTau indicates that the TOMA1-binding conformation is the synaptotoxic species in this recombinant tau preparation. Furthermore, the lack of efficacy against the disruptive action of SτAs confirms that the synaptotoxic species in SτAs has a different conformation from that of oTau. Indeed, there is growing antibody-based evidence of multiple soluble toxic tau assemblies in AD and other tauopathies [27, 53, 54]. In pilot studies, we found that soluble synaptotoxic tau-containing AD brain extract inhibited LTP in a manner that was resistant to TOMA1, leaving open the possibility that the oTau preparation only partly models the complexity of AD brain soluble tau.

Based on the present findings, at least two distinct tau assemblies likely act at distinct biological toxic receptors/acceptors to mediate the disruptive effects of SτAs and oTau on synaptic plasticity. Accumulating evidence over the last decade has shown that tau can be actively transported both out of and into glia and neurons via mechanisms that are not yet fully understood [16, 55–57]. Whether or not tau monomers, oligomers and fibrils are differentially handled by transporters is a matter of ongoing research [58, 59]. Clearly, the relative role of extracellular versus intracellular sites of action will depend on membrane transport. In the case of oTau, it has been reported to be readily transported into cells by an amyloid precursor protein (APP)-dependent process and has been posited to act primarily intracellularly to inhibit LTP [17]. Furthermore, recombinant tau oligomers (prepared by maleimide labelling) can have very different effects on synaptic function and plasticity when applied at different sites of neuronal cells [60]. At presynaptic sites the tau oligomers induced the run-down of unitary excitatory postsynaptic potentials, which was associated with increased short-term depression. In contrast, the introduction of the tau oligomers into postsynaptic neurons did not affect basal synaptic transmission but markedly impaired the induction of LTP [60]. SτAs are readily taken up by neurons [59] and thus are likely to exert intracellular actions. We published evidence that cellular prion protein (PrPc) may act as an extracellular receptor/acceptor to mediate both the inhibition of LTP and LTD by SτAs at doses that did not affect baseline synaptic transmission, paired-pulse facilitation or the response during high-frequency stimulation [38, 39]. To our surprise, on examining the synaptic responses during the 1 Hz conditioning stimulation we find that SτAs, at a dose that inhibits LTD, increased frequency facilitation (Supplemental Figs. 2a, b, Additional file 1). This 1 Hz facilitation, like the disruptive effects of SτAs on LTP and LTD [38], was inhibited by a GluN2B NMDA receptor antagonist (Supplemental Figs. 2c, d, Additional file 1), indicative of inappropriate activation of NMDA receptors. In contrast, a metabotropic glutamate receptor 5 (mGlu5R) antagonist mimicked and occluded the facilitatory effect of SτAs during the 1 Hz conditioning protocol (Supplemental Figs. 2e, f, Additional file 1). Moreover, oTau and TNFα were without observable effect (Supplemental Figs. 2 g-j, Additional file 1) as was synaptotoxic tau in aqueous brain extracts from people with AD or Pick’s disease tauopathy (Supplemental Figs. 3a-d, Additional file 1). The differential action of SτAs indicates an ability to recruit local circuits that do not appear to be affected in the same way by patient-derived synaptotoxic tau, oTau or TNFα. PrPc can act as a co-receptor for mGlu5Rs and thereby regulate NMDA receptor-mediated events in the presence of synaptotoxic Aß oligomers [39, 40]. Presumably because of complex activity-dependent mechanisms downstream of PrPc, the disruption of LTP, but not LTD, by SτAs is mGluR5-dependent [38]. Even though SτAs inhibit LTD and thus oppose activity-dependent synaptic weakening, they potently trigger synaptic loss by binding PrPc [39, 41].

In AD and other tauopathies, a variety of different soluble tau assemblies are likely to co-exist on a background of deposits of fibrillar tau in NFTs [61, 62]. Here, we examined the possibility that different soluble forms may interact when present together. We found that exogenously applied SτAs blocked the ability of oTau to facilitate LTD. This indicates that if similar levels of fibril-derived and monomer-derived soluble tau assemblies are co-located the action of the former tau species would predominate. The suppression of oTau-induced LTD facilitation by SτAs could be explained by several potential mechanisms including a direct interaction at the chemical level or a more systems-based interaction whereby the presence of SτAs might trigger cellular pathways which could override the pathways that mediate the ability of oTau to facilitate LTD. We previously reported that Aß oligomer-facilitated LTD is also blocked by SτAs [38]. In the present report we found that co-administration of SτAs prevented the ability of LFS300-1 Hz failed to induce LTD by TNF. Taken together, these findings indicate that the inhibition of oTau-facilitated LTD by SτAs is due to antagonism at the physiological rather than chemical level.

Misfolded proteins trigger multiple cellular defenses, including innate immune reactions, which can lose their protective functions when strongly activated. Recently, we reported that dampening down the pro-inflammatory cytokine TNFα with etanercept could partly ameliorate the enhancement of LTD by patient-derived synaptotoxic tau [48]. Here, we found that etanercept also attenuated the disruptive effect of oTau but not SτAs, supporting the proposal that oTau may better model these tau species than SτAs. Furthermore, we found preliminary evidence that oTau can increase the concentration of TNFα in the hippocampus (Supplemental Fig. 4, Additional file 1), consistent with previous reports that tau can trigger innate immune responses in the brain [63, 64] and in vivo imaging that tau brain levels are tightly correlated with microglial changes in early AD [65]. The finding that the inhibition of LTP and LTD by SτAs is insensitive to an agent that reduces a key inflammatory cytokine is consistent with the likely involvement of PrPc in mediating these deleterious synaptic effects. PrPc-mediated synaptotoxicity of Aß has been reported to be relatively independent of pro-inflammatory mechanisms [66].

Our finding that injection of TNFα alone also facilitated LTD induction by weak LFS-300 complements previous reports that genetic knockout of TNFR1, the main pro-inflammatory receptor for TNFα, can prevent LTD induced by a standard LFS-900 protocol [67, 68]. TNFα is elevated by multiple factors that mediate inflammaging, and we reported that patient-derived synaptotoxic tau facilitates LTD in an age-dependent manner that is linked to activation of the integrated stress response [48]. In pilot studies we tested the effect of oTau on LTD in middle-aged rats (Supplemental Figs. 5a, b, Additional file 1). Consistent with our previous report, a relatively low dose that didn’t affect LTD threshold in young rats facilitated LTD in the older animals. Thus, the ability of oTau to promote LTD in a TNF-dependent manner suggests a key role for this pro-inflammatory cytokine in synaptic weakening across a wide range of age-related neurodegenerative disorders [31]. It seems likely that TNFα acts in a pleiotropic manner to inappropriately modulate multiple pathways and thereby mediate metaplastic effects to inhibit LTP and facilitate LTD [69–71].

In conclusion, these findings highlight the intricate interplay between soluble tau assemblies, inflammatory cytokines, and synaptic plasticity. We propose that elucidating the means by which pathological tau impairs synaptic function provides a powerful means for exploring novel therapeutic strategies for AD. Based on our present and previous findings [39] the question arises as to how much different preparations of tau, either recombinant or AD patient-derived, containing varying amounts and forms of synaptic plasticity-disrupting tau are representative of tau assemblies present in AD brain. It will be critical to further investigate the physicochemical behavior of synaptotoxic tau in a wide range of patient-derived and recombinant protein preparations [13, 16, 37].

Furthermore, the ameliorating effect of the TNFα inhibitor etanercept on oTau-facilitated LTD lends support to the therapeutic potential of targeting inflammatory pathways to mitigate synaptic dysfunction in early tauopathies.

## Electronic supplementary material

Below is the link to the electronic supplementary material.

Additonal file 1

## Data Availability

The data that support the findings of this study can be made available upon reasonable request by contacting the corresponding author (MJR).
