# Supplementary material for: Divergent disruptive effects of soluble recombinant tau assemblies on synaptic plasticity in vivo
Source: Mol Brain. 2025 Apr 18;18:36. doi: 10.1186/s13041-025-01208-8 (PMC12007380; doi:10.1186/s13041-025-01208-8)
Supplement: Supplementary file 1 — Additonal file 1 [file 13041_2025_1208_MOESM1_ESM.pdf]

Additional file 1

Divergent disruptive effects of soluble recombinant tau assemblies on synaptic plasticity *in vivo*

Yin Yang[1,2 a]**,Tomas Ondrejcak**[2a]**, Neng-Wei Hu**[1,2]**, Igor Klyubin**[2]**, Michael J. Rowan**[2]

[1]Department of Physiology and Neurobiology, School of Basic Medical Sciences, Zhengzhou University, 100 Science Avenue, Zhengzhou 450001, China

[2]Department of Pharmacology & Therapeutics, School of Medicine, and Institute of Neuroscience, Trinity College, Dublin 2, Ireland

[a]*These authors contributed equally to the study*

Correspondence should be addressed to: Michael Rowan or Neng-Wei Hu, Pharmacology & Therapeutics, Watts Building, Trinity College Dublin, Dublin 2, Ireland. E-mail: mrowan@tcd.ie or hunw@tcd.ie

See https://doi.org/10.1186/s13041-025-01208-8

Supplementary Material

(1) Supplementary Figures 1-5
(2) Supplementary References

Supplementary Figures

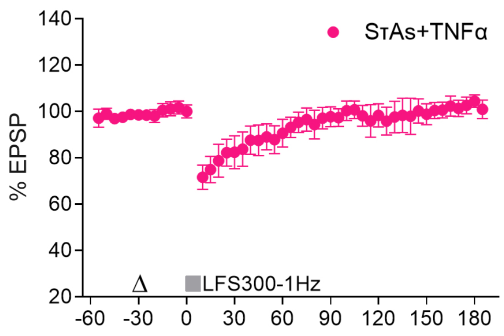

Supplementary Figure 1

Recombinant soluble tau aggregates (SτAs) prevent the facilitation of LTD by TNFα

The application of weak LFS (bar, LFS300-1Hz, 300 pulses at 1 Hz) did not induce LTD in rats receiving i.c.v. co-injection of SτAs (1.2 pmol) with the pro-inflammatory cytokine TNFα (1.5 pmol) (SτAs+TNFα, n=3). This dose of TNFα facilitates LTD in the absence of SτAs (see Figure 3 c,d in main text). Values are mean ± SEM.

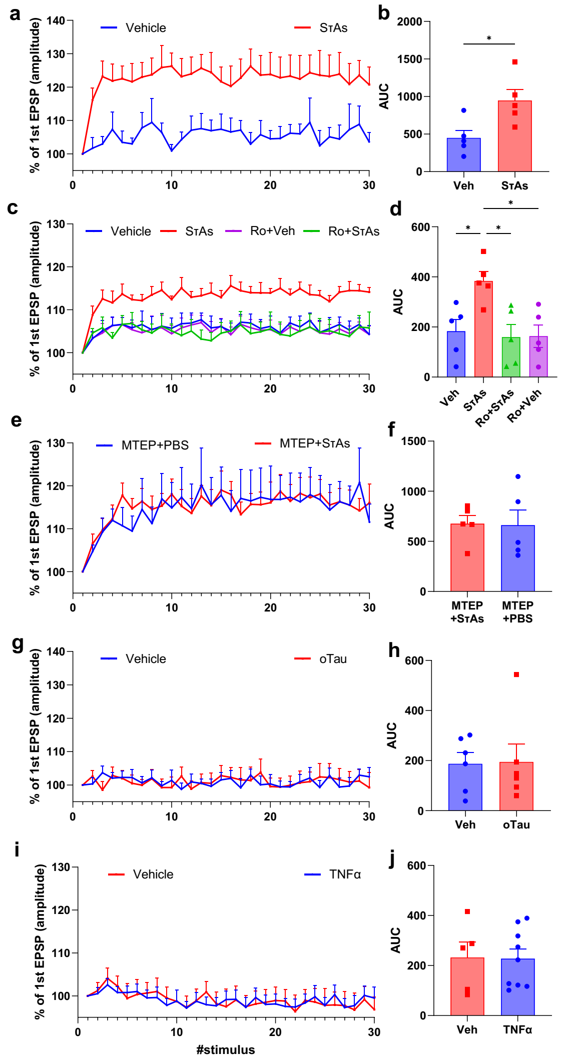

Supplementary Figure 2

Recombinant soluble tau aggregates (SτAs), but not oTau, enhance 1 Hz facilitation *in vivo*

Dorsal hippocampal evoked responses at CA1-to-CA3 synapses during the LFS (first 30 pulses at 1 Hz) are expressed as a percentage of the first fEPSP. Summary bar charts express the change in the 30 evoked responses as area under curve (AUC). Data in (a,b) and (g-j) refer to the relevant experimental groups shown in the main article. Data in (c-f) are further analyses of our previously published experiments (Ondrejcak *et al.* 2019). Frequency facilitation of fEPSPs recorded during the 1 Hz stimulation conditioning protocol used to induce LTD was enhanced by 0.6 pmol SτAs (**a-d**) and prevented by systemic pre-treatment with the GluN2B subunit-containing NMDAR antagonist Ro 25-6981 (12 mg/kg, i.p.) (**c,d**). Enhancement of 1Hz facilitation was also observed in animals treated with MTEP (a selective mGluR5 antagonist, 3m/kg, i.p.) which occluded the facilitatory effect of SτAs (**e,f**). Acute i.c.v. treatment with Vehicle; oTau (12.3pmol) or TNFα (1.5pmol) 15 min prior to 1 Hz stimulation did not cause any observable change in this form of short-term facilitation (**g-j**). All experiments were carried out under non-recovery urethane anaesthesia. Values are mean ± SEM. *$p < 0.05$

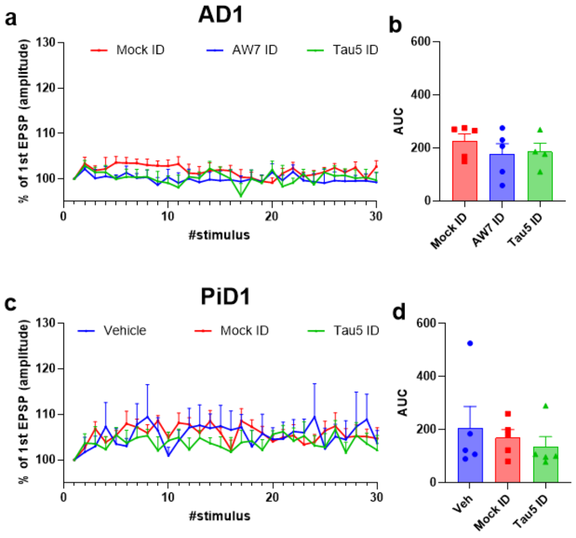

Supplementary Figure 3

Synaptotoxic tau in aqueous extracts of Alzheimer's disease and Pick's disease (PiD) tauopathy brains fails to significantly enhance 1 Hz facilitation *in vivo*

We previously reported that the Alzheimer's disease brain aqueous extract named AD1 facilitates hippocampal LTD in a tau-dependent manner (Hu *et al.* 2024). In that publication we found that i.c.v. injection of aqueous extract of this brain (either immunodepleted, ID, of Aß using the polyclonal antibody AW7, AW7 ID, or mock ID, Mock ID) facilitated the induction of a robust and persistent LTD by LFS-300 protocol. In contrast, in animals injected with this extract that had been ID of tau using the mid-region monoclonal antibody Tau 5 (Tau5 ID), LFS300-1Hz failed to enable the induction of LTD (Hu *et al.* 2024). Here we measured the fEPSPs during the 1Hz conditioning protocol used in those experiments. (**a,b**) Analysis of short-term plasticity in the form of frequency facilitation during the LFS300-1Hz protocol, indicated that injection of AD1 brain extract (Mock ID, AW7 ID or Tau5 ID) had no effect. (**c,d**) Similarly, acute i.c.v. injection of a Pick's disease (PiD1) aqueous brain extract containing LTP and LTD disrupting tau (Hu *et al.* 2024, Ondrejcak *et al.* 2024), that was mock immunodepleted (Mock ID) or ID of tau with the anti-tau antibody Tau5 (Tau 5 ID) did not appear to affect the synaptic responses during the 1 Hz conditioning stimulation (analysis based on published data in Hu *et al.* 2024). All experiments were carried out under non-recovery urethane anaesthesia. Values are mean + SEM.

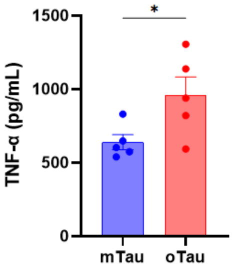

Supplementary Figure 4

oTau acutely increases the concentration of TNFα in rat hippocampus *in vivo*

Measurement of TNF-α levels in hippocampal homogenates after i.c.v. injection of 20 pmol mTau or oTau was performed with ELISA kit (ZC-37624, ZCi Bio, Shanghai, China) under non-recovery urethane anaesthesia. Brains were removed 3.5 h after mTau or oTau injection. Roughly 0.2 g of dorsal hippocampus tissue was collected and homogenized with 1 mL pre-cold PBS containing protease inhibitor cocktail (CW2200S, CWBIO, China). Samples were sonicated for 2 min with 170 W power. Repeated freezing and thawing was performed two times for additional cell membrane breakage. After centrifugation (5000 g at 4°C for 10min), supernatants were used for measurement and procedures were carried out following the kit instructions. Values are mean ± SEM. *$p < 0.05$

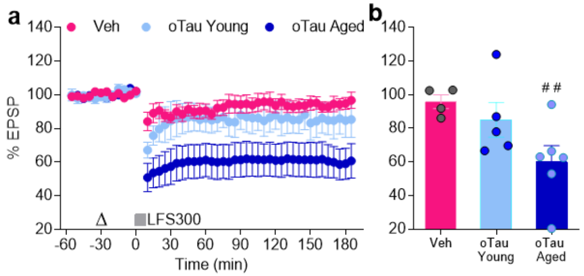

Supplementary Figure 5

The facilitation of LTD by oTau is enhanced in middle-aged rats

We previously reported that 12.3 pmol oTau inhibited LTP in young adult rats (Ondrejcak *et al.* 2024). This dose, which had no observable effect on baseline synaptic transmission also facilitated LTD induction by LFS300-1Hz (see main article). Here we examined the effect of age on the ability of oTau to facilitate LTD. (**a**,**b**) Whereas a relatively low dose (2.5 pmol, i.c.v. denoted by triangle symbol) of oTau had no effect in young rats it enabled the induction of LTD in middle-aged animals. All experiments were carried out under non-recovery urethane anaesthesia. These findings suggest that oTau facilitates LTD in an age-dependent manner. All experiments were carried out under non-recovery urethane anaesthesia. Summary bar charts in **b** show the magnitude of LTD during the last 10 min for the time-course data in **a**, respectively. Values are mean ± SEM. $^{\#\#}p < 0.01$ compared with pre-HFS baseline, paired *t*-test

Supplementary References

1. Hu, N. W., T. Ondrejcak*, et al.* (2024). "Patient-derived tau and Aβ facilitate LTD in vivo: role of TNFα and the integrated stress response." Brain Commun **6**(5): fcae333.
2. Ondrejcak, T., N.-W. Hu*, et al.* (2019). "Soluble tau aggregates inhibit synaptic long-term depression and amyloid β-facilitated LTD in vivo." Neurobiol Dis **127**: 582-590.
3. Ondrejcak, T., I. Klyubin*, et al.* (2024). "Rapidly reversible persistent LTP inhibition by patient-derived brain tau and amyloid β proteins." Phil. Trans. R. Soc. B **379**(20230234).
